# Examining a Remote Group-Based Type 2 Diabetes Self-Management Education Program in the COVID-19 Era Using the ORBIT Model: Small 6-Week Feasibility Study

**DOI:** 10.2196/46418

**Published:** 2024-01-29

**Authors:** Madison S Hiemstra, Sonja M Reichert, Marc S Mitchell

**Affiliations:** 1 School of Kinesiology Western University London, ON Canada; 2 Centre for Studies in Family Medicine Department of Family Medicine Schulich School of Medicine and Dentistry, Western University London, ON Canada

**Keywords:** activity monitor, diabetes self-management education, flash glucose monitor, glycated hemoglobin, group education, HbA1c, T2D, type 2 diabetes, virtual care, wearables

## Abstract

**Background:**

To date, most group-based diabetes self-management education (DSME) programs for type 2 diabetes (T2D) have been delivered in person. The rapid transition to remote care at the outset of the COVID-19 pandemic presented opportunities to test, evaluate, and iterate a new remote DSME program.

**Objective:**

We aim to refine the delivery and evaluation of a multicomponent remote DSME program for adults living with T2D by examining several feasibility outcomes.

**Methods:**

We recruited a convenience sample of patients from a London, Canada, outpatient diabetes clinic (serving high-risk, low-income adults) to participate in a 6-week, single cohort feasibility study from November 2020 to March 2021. This small ORBIT phase 1b feasibility study represents the first in a planned series guided by the ORBIT model for developing behavioral interventions for chronic diseases (phase 1: design; phase 2: preliminary testing; phase 3: efficacy; and phase 4: effectiveness). The feasibility of delivering and evaluating a remote DSME program, including (1) live video education classes, (2) individualized physical activity (PA) prescription and counseling, and (3) intermittently scanned continuous glucose and wearable PA monitoring, was assessed. Feasibility outcomes included recruitment and retention rates, program adherence, and acceptability (ie, technology issues and exit survey feedback). PA was assessed with Fitbit Inspire 2 (Fitbit Inc) and estimated glycated hemoglobin (HbA_1c_) using the FreeStyle Libre (Abbot). Given the small study sample, group- and individual-level data are reported descriptively.

**Results:**

A total of 10 adults living with T2D were recruited (female 60%; age 49.9, SD 14.3 years; estimated HbA_1c_ 6.2%, SD 0.5%). Recruitment and retention rates were 29% and 80%, respectively. Participants attended 83% (25/30) and 93% (37/40) of education classes and PA counseling phone calls, respectively. There were 3.2 (SD 2.6) technology issues reported per person, most of which were related to study data transfer. Exit survey responses suggest most participants (8/9, 89%) were “satisfied” with the program. Recognizing the small sample size and the fact that no inferential statistics were conducted, the mean (SD) for the weekly daily step count and estimated HbA_1c_ are provided for illustrative purposes. Participants accumulated 7103 (SD 2900) and 7515 (SD 3169) steps per day at baseline and week 6, respectively. The estimated HbA_1c_ was 6.2% (SD 0.5%) and 6.2% (SD 0.6%) at baseline and week 6, respectively.

**Conclusions:**

This ORBIT phase 1b study served to refine the delivery (eg, automatic study data upload process recommended to reduce participant burden) and evaluation (eg, purposeful sampling of participants with baseline HbA_1c_ >8% recommended to address selection bias) of a remote DSME program. Preliminary proof-of-concept testing (ORBIT phase 2) incorporating some of these learnings is now warranted.

**Trial Registration:**

ClinicalTrials.gov NCT04498819; https://clinicaltrials.gov/study/NCT04498819

## Introduction

Chronic conditions such as type 2 diabetes (T2D) are best treated when the individual living with diabetes is engaged and supported in effective self-management [1,2]. Programs, such as diabetes self-management education (DSME), that promote successful T2D self-management behaviors can dramatically lower the risk of serious complications [2]. Group DSME programs promote sustained self-care [2]; however, in response to the COVID-19 pandemic, most group DSME programs were quickly transitioned to remote, one-on-one delivery [3]. To support this rapid transition, contemporary technologies were thrust into the forefront, including videoconferencing services (eg, WebEx and Cisco), intermittently scanned continuous glucose monitors (eg, FreeStyle Libre and Abbott’s Diabetes Care Division), and wearable activity trackers (eg, Fitbit) [3]. This preparatory study examined the feasibility of delivering and evaluating remote group DSME programming for adults with T2D in the COVID-19 era. This small cohort study, aligning with phase 1b of the ORBIT model for developing behavioral treatments for chronic diseases [4], is the first in a planned series aiming to systematically develop an efficacious remote group DSME program for broad rollout in Canada (the “LIBERATE” program [5]). The central goal of this work is to refine the delivery and evaluation of a remote group DSME program (the “treatment”) to promote efficiency while producing potentially relevant changes in behavioral (eg, physical activity [PA]) and clinical risk factors (eg, glycemic control) [4].

## Methods

### Overview

A single-arm feasibility study (ORBIT phase 1b) was conducted between November 2020 and March 2021 in London, Ontario. Ontario COVID-19–related physical distancing policies were in place throughout the study period, with strict stay-at-home orders for 9 out of 16 weeks [6]. Despite well-documented pandemic-related recruitment challenges [7], we sought to recruit a convenience sample of 10 to 20 new patient intakes [8-12] from St Joseph’s Primary Care Diabetes Support Program, a London, Ontario, diabetes outpatient clinic serving higher-risk, lower-income adults [13]. Prospective participants, physician-cleared to exercise and with access to a smartphone (ie, iPhone 7 iOS or higher or Android [operating system 5 or higher]), were invited to a study recruitment session by their physicians during usual care.

### Intervention

A 6-week remote group DSME program was delivered to participants between November 2020 and March 2021. A rolling intake was used, with participants completing the program over 6 consecutive weeks. The program included (1) live-video delivery (ie, WebEx) of biweekly group education classes by a multidisciplinary team (ie, physician, diabetes nurse educator, and exercise specialist) and grounded in *Diabetes Canada*’s Self-Management Education Guidelines [2]; (2) biweekly one-on-one PA counseling phone calls; and (3) enhanced self-monitoring using intermittently scanned continuous glucose (FreeStyle Libre; Abbot) and wrist-worn PA (Fitbit Inspire 2; Fitbit Inc) monitors. The Inspire 2 was selected as it was the most affordable Fitbit model, offering features required for the study (eg, daily step tracking and data exporting). Group education classes and PA counseling calls were designed to help participants learn from the biofeedback they were receiving (eg, draw important linkages between FreeStyle Libre-assessed glucose trends, diet, and PA behaviors) [14]. Fitbit data were used to generate individualized and adaptive daily step count goals [15] (ie, daily step count median from the past 14 days + 500 steps, equivalent to 5 more minutes of brisk walking [16]). The exercise specialist reviewed step goals with participants during biweekly PA counseling calls. Participants were instructed to submit Fitbit data (by downloading a Fitbit Excel file and uploading it to a secure file sharing website) and scan their FreeStyle Libre frequently.

### Outcomes

The primary study objective was to refine the delivery and evaluation of the remote group DSME program in preparation for an ORBIT phase 2a study (preliminary testing: proof-of-concept). To do this, (1) recruitment and retention, (2) program adherence (ie, attendance, Fitbit data submission rates, and FreeStyle Libre data capture rates [active time]), and (3) acceptability (ie, number of technology issues reported and exit survey responses) data were collected. “Active time” is the mean biweekly percent of total glucose data captured by the FreeStyle Libre every 24 hours. To examine the potential impact of the program on behavioral and clinical risk factors, device-assessed (4) weekly mean daily step count and (5) estimated glycated hemoglobin (HbA_1c_; the average glucose level from the FreeStyle Libre readings for 14 or more days [17]) were collected.

### Analysis

Given the small, single cohort and preparatory nature of this ORBIT phase 1b feasibility study, individual and group-level data are presented descriptively rather than with inferential statistics.

### Ethical Considerations

This study was registered at ClinicalTrials.gov (NCT04498819) and approved by Western University’s Health Science Research Ethics Board (116071). Patients provided informed consent before participating. Deidentified study data were stored on Western University’s password-protected and encrypted OneDrive (Microsoft Corporation).

## Results

Among 35 eligible new patient intakes, 10 were enrolled, meeting our a priori recruitment target (Table 1; 29% recruitment rate). Reasons for nonparticipation (n=5) included work-time conflict, a sick partner, being too busy, not wanting to wear the FreeStyle Libre, and feeling exercise and nutrition were well-managed. A total of 2 participants dropped out of the study (ie, participant 1 missed 2 consecutive biweekly group education classes, and participant 8 withdrew during week 5 citing lack of time). A total of 8 participants completed follow-up assessments (80% retention). Regarding program adherence, 83% (25/30) and 93% (37/40) of participants attended group education classes and one-on-one PA counseling phone calls, respectively. Additionally, 53% (16/30) of participants submitted Fitbit data, and the FreeStyle Libre captured 81% of blood glucose data during the intervention period (“Active time”). FreeStyle Libre use may have waned after week 4, as 4 out of 8 (50%) participants had their lowest data capture rates in weeks 5 and 6 (Table 2).

**Table 1:** Participants’ characteristics.

			Value
**Sociodemographics**			
	Age (years), mean (SD); range		49.9 (14.3); 36-73
	Sex (female), n (%)		6 (60)
	Ethnicity (White), n (%)		4 (40)
	**Highest education level, n (%)**		
		Less than high school	2 (20)
		High school diploma or equivalent	4 (40)
		College certificate, university, or higher	4 (40)
	Employment status (employed full time), n (%)		5 (50)
	Household income (<US $39,277.30), n (%)		5 (55.5)
	Car ownership, n (%)		8 (80)
	**Relationship status, n (%)**		
		Single	3 (30)
		Married or equivalent	3 (30)
		Separated, divorced, or equivalent	3 (30)
		Widowed	1 (10)
**Health characteristics**			
	Years since diabetes diagnosis, mean (SD)		2.6 (3.3)
	Estimated baseline glycated hemoglobin (%), mean (SD)		6.2 (0.5)
	Systolic blood pressure (mmHg), mean (SD)		131 (16.7)
	Diastolic blood pressure (mmHg), mean (SD)		79.4 (7.9)
	**Comorbidities, n (%)**		
		Dyslipidemia	6 (60)
		Hypertension	4 (40)
		Anxiety	3 (30)
		Depression	2 (20)
		Other psychiatric condition	2 (20)
		Chronic kidney disease	3 (30)
		Heart disease^a^	3 (30)
	Baseline physical activity (steps per day), mean (SD)		7103 (2900)

^a^Including coronary artery disease, heart failure, or arrythmia.

**Table 2:** Biweekly mean estimated glycated hemoglobin (HbA1c) and “Active time” by participant and for the total sample.

			Baseline				Weeks 1-2					Weeks 3-4					Weeks 5-6		
			Estimated HbA_1c_^a^		Active time^b^	Estimated HbA_1c_^a^			Active time^b^		Estimated HbA_1c_^a^			Active time^b^		Estimated HbA_1c_^a^			Active time^b^
**By participant (%), mean**																			
	1^c^	N/A^d^		34		N/A		35		N/A			N/A		N/A			N/A	
	2^c^	6.0		99		6.0		94		6.2			99		6.3			98	
	3^c^	N/A		52		6.7		67		6.4			64		6.3			70	
	4^c^	6.1		83		6.0		85		5.8			88		5.8			81	
	5^c^	7.0		89		7.0		94		7.3			83		6.9			77	
	6^c^	5.6		97		5.6		98		5.5			100		5.3			90	
	7^c^	N/A		95		N/A		100		N/A			100		N/A			100	
	8^c^	6.2		89		6.2		87		6.5			93		N/A			N/A	
	9^c^	6.7		87		7.0		84		7.4			88		6.8			86	
	10^c^	5.8		66		5.7		50		6.2			56		N/A			37	
Total (%), mean (SD)			6.2 (0.5)		77 (22)	6.3 (0.6)			77 (22)		6.4 (0.7)			84 (16)		6.2 (0.6)			77 (20)

^a^The estimated HbA_1c_ levels are percentages as measured by the FreeStyle Libre.

^b^Active time is the mean percentage of total blood glucose data captured in a 24-hour period.

^c^Participant identification number.

^d^N/A: not applicable.

Participants reported at least 1 technology issue, with 3.2 (SD 2.6) issues reported per person, including difficulties submitting Fitbit data (n=14), lost WebEx remote group education class link (n=6), FreeStyle Libre monitor falling off prematurely (n=7 of 32 sensors distributed in total), difficulty synchronizing Fitbit with participant smartphone (n=3), and losing a Fitbit altogether (n=2). Exit survey responses (Table S1 in Multimedia Appendix 1) suggested participants were satisfied with the program, with most (8/9, 89%) agreeing with the statement, “Overall, I was satisfied with the program.” Exit survey responses also indicated that combined biofeedback from FreeStyle Libre and Fitbit (4/9, 44%) was most helpful in optimizing self-management. Daily step counts for the total sample were 7103 (SD 2900) steps and 7515 (SD 3169) steps at baseline and week 6, respectively (Table 3). Lastly, estimated HbA_1c_ was 6.2% (SD 0.5%) and 6.2% (SD 0.6%) at baseline and week 6, respectively (Table 2).

**Table 3:** Biweekly daily step count, in mean (SD), by participant and for the total sample.

		Baseline, mean (SD)	Weeks 1-2, mean (SD)	Weeks 3-4, mean (SD)	Weeks 5-6, mean (SD)
**By participant**					
	1^a^	N/A^b^	N/A	N/A	N/A
	2^a^	9729 (2985)	11,158 (3917)	9521 (4002)	8165 (5222)
	3^a^	2114 (663)	1790 (585)	1779 (948)	2103 (746)
	4^a^	9191 (2794)	8001 (1800)	9187 (1654)	8987 (2203)
	5^a^	6112 (1762)	6154 (1590)	5052 (1526)	5487 (1823)
	6^a^	8954 (2569)	8979 (1890)	10,286 (1279)	10,111 (1744)
	7^a^	11,114 (3595)	13,114 (1736)	12,484 (1743)	12,930 (2619)
	8^a^	4877 (1733)	4355 (1212)	N/A	N/A
	9^a^	4721 (2063)	5113 (1195)	5366 (2177)	6511 (2936)
	10^a^	7115 (2622)	7570 (2741)	6786 (1999)	6634 (4062)
Total		7103 (2900)	7359 (3485)	7558 (3449)	7515 (3169)

^a^Participant identification number.

^b^N/A: not applicable.

## Discussion

### Overview

The aim of this study was to refine the delivery and evaluation of a remote group DSME program in preparation for an ORBIT phase 2a study (preliminary testing: proof-of-concept). This DSME program showed promise, albeit with a small convenience sample, with most participants being satisfied with the program. Fitbit and FreeStyle Libre data capture was also high (>80% in both cases). Moreover, preliminary data suggest potential for behavioral risk factor (ie, PA) improvement over a short 6-week period. However, several opportunities to improve the protocol were identified (Table 4). These should be addressed before moving onto the ORBIT phase 2a study*.* For instance, reducing participant burden by offering automatic study data upload may increase evaluation and program efficiency. Additionally, purposefully sampling participants with higher baseline HbA_1c_ (ie, HbA_1c_>8%) [18] may help address selection bias and the “floor effect” [19] in the future (eg, T2D was generally well-controlled among participants at baseline).

The findings should be considered in light of similar research. Our 29% recruitment rate, for example, was comparable to recruitment rates of 21% to 78% in similar studies [10,11,20-22]. Suboptimal recruitment rates could be attributed to (1) limited physician referrals to the study recruitment session, (2) additional participant stressors amid the COVID-19 pandemic (ie, employment insecurity), (3) seasonal effects (ie, colder winter months) [23], or (4) perceived study burden. Technology access and cost-related barriers did not appear to limit participation in this lower-income population [21]. High program adherence and study retention (>80%) suggest remote delivery of the DSME program was generally well-received in this small convenience sample, aligning with a modest but growing number of studies in this field (eg, remote group education class attendance in similar studies has ranged from 52% to 95%) [10,20].

This study is not without limitations. First, our sample was small, consisting of participants with well-controlled T2D (perhaps not representative of those who may benefit most from DSME). However, given the sample sizes of comparable studies [10,11,24] and the objectives of this preparatory work, we contend it is appropriate given the conclusions being drawn. Future recruitment rates may improve with the easing of COVID-19-related restrictions [25] and as we embark on year-round recruitment in the next study in this planned series. Second, this program was only 6 weeks long, providing little insight into sustained program adherence in a field where attrition is the norm [26]. Third, this program was resource-intensive (eg, required significant staff time). Scaling the program (ie, having monthly classes after the first 6 weeks) to reduce resource requirements for a longer program may be necessary. Lastly, discrete exit survey responses only provided so much insight (eg, compared to conducting focus groups [4,9]). Moving ahead to ORBIT phase 2a, focus groups will be conducted to gather deeper insights.

**Table 4:** Study protocol areas for improvement and recommendations.

Protocol	Areas for Improvement	Recommendations
Recruitment	Low recruitment rate (29%), likely due to a low referral rate to study recruitment sessions by clinicians or the colder winter season Potentially high participant burden	Clearer recruitment instructions for clinicians Recruit year-round for the ORBIT phase 2a trial Streamline recruitment procedures (eg, in-person sign-up for study recruitment sessions, etc)
Sample	Possible selection bias with generally well-controlled diabetes (baseline estimated HbA_1c_^a^=6.2%) and technology as participation barriers Both insulin and noninsulin users are included	Purposeful sampling to include baseline estimated HbA_1c_ >8% and individuals who do not have ready smartphone access Conduct subanalyses of insulin versus noninsulin users
Technology	Issues (14 total) with study data submissions Remote classes sound-related “feedback” issue Early evidence of FreeStyle Libre attrition, with lower “active time”^b^ observed for some participants in Weeks 5 and 6	Provide the option to email or text a screenshot of the 2-week Fitbit step count summary or implement automatic data upload Ensure class facilitators have “mute” capabilities Create short booster sessions to encourage exploration of personal FreeStyle Libre data and shared experiences
Group class	Limited group discussions leading to more didactic education Difficulty “reading the room” when telephoning into remote classes Rolling intake format	Emphasize the importance of sharing and peer learning; provide 1-page content summaries to review before class; and provide assignments to reinforce learning Encourage participation through a video platform with the camera on or off Remove the rolling intake. Instead, have set start and end dates
Physical activity	Daily step count prescriptions only. Participants had a choice on “how” to accumulate the steps Cardiovascular fitness outcomes were not assessed A daily step count of 500 steps or more was considered a full day worth of data (valid day)	Offer a choice of time, type, and frequency of a preferred exercise (to compliment the daily step count goal); increase the frequency and automation of feedback Use validated field test measures to assess cardiovascular fitness level changes (eg, a 6-minute walk test) A “valid day” may include the time between the first and last daily step recorded

^a^HbA_1c_: glycated hemoglobin; an estimation provided from 2 weeks or more of glucose data collected by the FreeStyle Libre.

^b^Active time is the mean percentage (%) of total glucose data captured in a 24-hour period every two weeks (the FreeStyle Libre requires at least 1 scan every 8 hours to collect the past 8 hours of data).

### Conclusions

This ORBIT phase 1b study has served to refine the delivery and evaluation of this remote DSME program. Proof-of-concept testing is warranted, with plans to progress to ORBIT model phases 3 (efficacy) and 4 (effectiveness). This may ultimately increase access to strong remote-group DSME programming in Canada.

## Appendix

Multimedia Appendix 1 Exit survey results.

## Abbreviations

DSME: diabetes self-management education
HbA1c: glycated hemoglobin
PA: physical activity
T2D: type 2 diabetes

## Data Availability

The data sets generated or analyzed during this study are available from the corresponding author upon reasonable request.
