# Supplementary material for: Examining a Remote Group-Based Type 2 Diabetes Self-Management Education Program in the COVID-19 Era Using the ORBIT Model: Small 6-Week Feasibility Study
Source: JMIR Form Res. 2024 Jan 29;8:e46418. doi: 10.2196/46418 (PMC10862237; doi:10.2196/46418)
Supplement: Multimedia Appendix 1 [file formative_v8i1e46418_app1.docx]

**Appendix A**

Table 1. Exit survey results.

| Main Reason for Participating (%) (n=9) | **Get a FitBit** | **Get a Libre** | | **Individualized Exercise Prescription** | **Thought it would improve health** | | **Participating in studies** | **COVID-19 alternative opportunity** | | **Other** |
| --- | --- | --- | --- | --- | --- | --- | --- | --- | --- | --- |
|  | 0 | 11.1 | | 11.1 | 55.6 | | 11.1 | 0 | | 11.1 |
| Which part of the study MOTIVATED you the MOST to increase your physical activity? (%) (n=8) | **FitBit** | | | **Libre** | **Individualized Exercise Prescription** | | **Group Education Classes** | **1:1 Phone call with exercise specialist** | | |
|  | 25 | | | 37.5 | 12.5 | | 25 | 0 | | |
| The use of my Libre guided my decisions about.... (SELECT ALL THAT APPLY) (%) (n=9) | **Food** | | | **Exercise** | **Medication** | | **None** | **Other** | | |
|  | 100 | | | 77.8 | 33.3 | | 0 | 11.1 | | |
| Which part of the program helped you LEARN the MOST about how to better manage your diabetes? (%) (n=9) | **Combined FitBit and FreeStyle Libre information** | | | **Information from the FreeStyle Libre** | **Information from the FitBit** | | **Group education classes** | **1:1 phone calls with exercise specialist** | | |
|  | 44.4 | | | 11.1 | 0 | | 33.3 | 11.1 | | |
|  | | | **Strongly Disagree (%)** | | | **Disagree (%)** | **Neutral (%)** | **Agree (%)** | **Strongly Agree (%)** | |
| The option of using the FitBit to track my exercise was a great motivational tool (n=9) | | | 11.1 | | | 0 | 22.2 | 22.2 | 44.4 | |
| The option of using the Libre to track my glucose was a great motivational tool (e.g., eating, exercising) (n=9) | | | 11.1 | | | 0 | 11.1 | 22.2 | 55.6 | |
| I felt comfortable speaking in the group-based educational sessions (n=9) | | | 11.1 | | | 11.1 | 44.4 | 11.1 | 22.2 | |
| I had plenty of opportunity to ask questions during the group-based educational sessions (n=9) | | | 11.1 | | | 0 | 22.2 | 33.3 | 33.3 | |
| I felt encouraged by others in the group-based educational sessions (n=9) | | | 11.1 | | | 22.2 | 22.2 | 22.2 | 22.2 | |
| I would have liked to have a friend or family member join the group-based educational sessions (n=9) | | | 0 | | | 33.3 | 0 | 0 | 33.3 | |
| Technology: It was easy for me to join the group-based educational sessions (n=9) | | | 0 | | | 0 | 0 | 66.7 | 33.3 | |
| Technology: Throughout the study I felt like my personal health information was protected (e.g., my privacy was being upheld) (n=9) | | | 11.1 | | | 11.1 | 0 | 11.1 | 66.7 | |
| Overall, I would say this program helped me become more physically active than I was before (n=9) | | | 0 | | | 0 | 22.2 | 33.3 | 44.4 | |
| Overall, I was satisfied with the program (n=9) | | | 0 | | | 0 | 11.1 | 44.4 | 44.4 | |
